# Predicting potentially fatal conductive disease in sarcoidosis; can cardiac MRI define the risk?

**DOI:** 10.1186/1532-429X-15-S1-P66

**Published:** 2013-01-30

**Authors:** Brian Staub, Robert W Biederman

**Affiliations:** 1 Cardiac MRI, Allegheny General Hospital, Pittsburgh, PA, USA

## Background

Sarcoidosis incidence is approximately 1:135,000 Americans. Cardiac involvement triggers multiple conduction disturbances, the most severe being complete heart block and VT/VF. Non-caseating granulomas have been found in the myocardium in up to 50% of the cases of fatal sarcoidosis, and 67% of patients that died from cardiac causes had myocardial involvement. Late gadolinium enhancement cardiovascular MCMRRI (LGE) has been shown to be more than twice as sensitive for cardiac involvement per current consensus Japanese Ministry of Health criteria and is widely held but, as yet unproven, to be more sensitive than the invasive RV endomyocardial biopsy.

## Methods

A retrospective single center study was performed by searching our cardiac MRI database for patients with pulmonary sarcoidosis and possible cardiac involvement. These patients' electronic medical records were then evaluated for documented history of complete heart block, ventricular arrhythmias and pacemaker and/or AICD implantation. Using the keyword "cardiac sarcoidosis", out of more than 6,500 clinical cardiac MRIs performed from 2001-2012 at our institution, 118 patients were identified, 58 of whom had evidence of pulmonary sarcoidosis (25 by biopsy, 12 by CT, 15 by MRI, and 6 by history).

## Results

Out of >6500 pts, 118 were referree for sarcoid. LGE identified additional cardiac involvement in 21 patients (36%). Five pts had documented VT while three pts had complete heart block (CHB). All eight had an AICD/PM placed. In the other 37 pts without cardiac involvement on LGE, only 1 pt required AICD placed for a history of CHB (p<0.05). LGE-defined cardiac involvement led to a significant prediction for complete heart block and VT/VF (p<0.001) as compared to more standard metrics.

## Conclusions

Patients with non-invasive LGE-CMR identified cardiac involvement have high potential to develop complete heart block/ventricular arrhythmias. Propensity risks suggest LGE may identify unstable electrical myocardium in a subset of patients warranting prophylactic AICD/PM placement. LGE defined yearly risk estimate for cardiac sarcoid (36%) is far greater than current post-implant AICD firing rate in ischemic CMX (<1%) at one year.

## Funding

None

